# *PTEN* loss is associated with prostate cancer recurrence and alterations in tumor DNA methylation profiles

**DOI:** 10.18632/oncotarget.20940

**Published:** 2017-09-15

**Authors:** Milan S. Geybels, Min Fang, Jonathan L. Wright, Xiaoyu Qu, Marina Bibikova, Brandy Klotzle, Jian-Bing Fan, Ziding Feng, Elaine A. Ostrander, Peter S. Nelson, Janet L. Stanford

**Affiliations:** ^1^ Department of Epidemiology, GROW School for Oncology and Developmental Biology, Maastricht University, Maastricht, The Netherlands; ^2^ Division of Public Health Sciences, Fred Hutchison Cancer Research Center, Seattle, Washington, USA; ^3^ Division of Clinical Research, Fred Hutchinson Cancer Research Center, Seattle, Washington, USA; ^4^ Department of Urology, University of Washington School of Medicine, Seattle, Washington, USA; ^5^ Department of Cytogenetics, Seattle Cancer Care Alliance, Seattle, Washington, USA; ^6^ Department of Oncology, Illumina, Inc., San Diego, California, USA; ^7^ Department of Biostatistics, MD Anderson Cancer Center, Houston, Texas, USA; ^8^ Cancer Genetics and Comparative Genomics Branch, National Human Genome Research Institute, NIH, Bethesda, Maryland, USA; ^9^ Division of Human Biology, Fred Hutchinson Cancer Research Center, Seattle, Washington, USA; ^10^ Department of Medicine, University of Washington School of Medicine, Seattle, Washington, USA; ^11^ Department of Epidemiology, University of Washington School of Public Health, Seattle, Washington, USA; ^12^ Current address: AnchorDx Corp., Guangzhou 510300, People's Republic of China

**Keywords:** phosphatase with tensin homology, epigenetics, prostate tumor methylation, recurrence and prognosis

## Abstract

**Background:**

Prostate cancer (PCa) with loss of the tumor suppressor gene *PTEN* has an unfavorable prognosis. DNA methylation profiles associated with *PTEN* loss may provide further insights into the mechanisms underlying these more aggressive, clinically relevant tumors.

**Methods:**

The cohort included patients with clinically localized PCa. Samples taken from the primary tumor were used to determine *PTEN* genomic deletions using FISH, and to analyze epigenome-wide DNA methylation profiles. Patients were followed for PCa recurrence on average for 8 years after diagnosis.

**Results:**

The study included 471 patients with data on *PTEN* loss, and the frequency of hemi- and homozygous *PTEN* loss was 10.0% and 4.5%, respectively. Loss of *PTEN* was associated with a significantly higher risk of recurrence (any vs. no *PTEN* loss; HR = 1.74; 95% CI: 1.03–2.93). Hazard ratios for hemi- and homozygous loss were 1.39 (95% CI: 0.73–2.64) and 2.84 (95% CI: 1.30–6.19), respectively. Epigenome-wide methylation profiling identified 4,208 differentially methylated CpGs (FDR Q-value < 0.01) in tumors with any versus no *PTEN* loss. There were no genome-wide significant differentially methylated CpGs in homo- versus hemizygous deleted tumors. Tumor methylation data were used to build a methylation signature of *PTEN* loss in our cohort, which was confirmed in TCGA, and included CpGs in *ATP11A*, *GDNF*, *JAK1*, *JAM3*, and *VAPA*.

**Conclusion:**

Loss of *PTEN* was positively associated with PCa recurrence. Prostate tumors with *PTEN* loss harbor a distinct methylation signature, and these aberrantly methylated CpG sites may mediate tumor progression when *PTEN* is deleted.

## INTRODUCTION

Phosphatase with tensin homology (*PTEN*) is one of the most frequently inactivated tumor suppressor genes in human cancers [1]. *PTEN* controls phosphoinositide 3-kinase (PI3K) signaling, which has critical roles in diverse cellular functions [2, 3]. As such, *PTEN* is involved in cell proliferation and survival, energy metabolism, and cellular architecture [4].

In PCa, loss of *PTEN* has been consistently associated with more aggressive disease features and a worse prognosis [5–16]. Estimates of *PTEN* loss range from less than 20 percent for clinically localized prostate tumors to more than 40 percent for metastatic castrate-resistant PCa [14, 17]. *PTEN* loss also frequently co-occurs with the *TMPRSS2:ERG* gene fusion, which exists in about half of all localized prostate tumors in men of European ancestry, suggesting that both somatic events might cooperate in prostate tumorigenesis [18, 19]. Loss of *PTEN* is now recognized as one of the major driving events in PCa [20].

Tumors with *PTEN* loss have significantly altered gene expression profiles. Saal *et al.* previously generated a tumor transcriptomic signature of *PTEN* loss in breast tumors [21]. The signature included 246 genes and the most significant differentially expressed gene was *PTEN* itself. The *PTEN* loss-like mRNA expression signature was also strongly associated with *PTEN* status based on copy number levels in PCa [22]. Further, in independent datasets of breast, prostate, and bladder carcinoma, the signature significantly correlated with worse patient outcomes [21].

Tumor epigenomic changes, in particular changes at the DNA methylation level, may also contribute to the progression of PCa [23]. Several studies have identified individual differentially methylated CpG sites and methylation signatures (combination of CpGs) associated with PCa recurrence and metastatic progression [24–26]. Recent research on PCa also revealed that methylation patterns are more tightly associated with patient outcomes than other genomic characteristics (e.g., copy number alterations, single nucleotide variants) [27].

Therefore, differential DNA methylation profiles in prostate tumors with *PTEN* loss may help to better understand the mechanisms that drive cancer progression in the absence of *PTEN*. To gain insights on this issue, we examined the association of *PTEN* loss with PCa recurrence in a cohort of patients diagnosed with clinically localized disease, and used methylome data from the same cohort to profile tumors with and without *PTEN* loss. As far as we know, this is the first large study to investigate *PTEN* status according to prostate cancer recurrence and epigenome-wide changes in tumor DNA methylation profiles.

## RESULTS

### Patient characteristics

There were 403 patients (85.6%) with *PTEN* intact tumors, and 47 (10.0%) and 21 (4.5%) patients with hemi- and homozygous *PTEN* loss, respectively (Table 1). *PTEN* loss was associated with higher Gleason scores (P = 0.03) and regional pathological stage (P < 0.01) as well as occurrence of the somatic *TMPRSS2:ERG* gene fusion (P < 0.01). There was no significant association between PTEN loss and race (European-American vs. African American; P = 0.4), but there were only 2 African-American patients with hemizygous PTEN loss and 3 African- American patients with homozygous PTEN loss.

**Table 1:** Selected patient characteristics by tumor *PTEN* status in the radical prostatectomy patient cohort^a^

	*PTEN* status									
	Intact (n = 403)			Hemizygous loss (n = 47)			Homozygous loss (n = 21)			
	No.	%	Mean (SD)	No.	%	Mean (SD)	No.	%	Mean (SD)	P-value^b^
Age at diagnosis (years)			58.4 (7.2)			57.3 (6.6)			56.2 (7.2)	0.10
Race										0.36
Caucasian	367	91.1%		45	95.7%		18	85.7%		
African-American	36	8.9%		2	4.3%		3	14.3%		
Pathological stage^c^										< 0.01
Local	295	73.2%		31	66.0%		8	38.1%		
Regional	108	26.8%		16	34.0%		13	61.9%		
Gleason score										0.03
≤6	207	51.4%		21	44.7%		5	23.8%		
7(3+4)	141	35.0%		13	27.7%		12	57.1%		
7(4+3)	30	7.4%		6	12.8%		3	14.3%		
8–10	25	6.2%		7	14.9%		1	4.8%		
PSA at diagnosis (ng/mL)										0.13
0–3.9	65	17.1%		8	17.0%		3	16.7%		
4–9.9	248	65.3%		25	53.2%		11	61.1%		
10–19.9	49	12.9%		8	17.0%		3	16.7%		
≥20	18	4.7%		6	12.8%		1	5.6%		
*TMPRSS2:ERG* fusion status										< 0.01
Negative	192	50.3%		14	29.8%		1	4.8%		
Positive	190	49.7%		33	70.2%		20	95.2%		
Recurrence										0.04
No	256	78.8%		26	70.3%		8	53.3%		
Yes^d^	69	21.2%		11	29.7%		7	46.7%		

^a^ Ninety-three patients had missing data on *PTEN* status. Patient characteristics were not substantially different for these patients.

^b^ P-value from either a T-test or chi-square test

^c^ Local: pT2, N0/NX, M0; regional: pT3–T4 and/or N1, M0

^d^ Of the patients with PCa recurrence, 17 had metastatic-lethal progression.

### *PTEN* loss and prostate cancer recurrence

**Figure 1:**
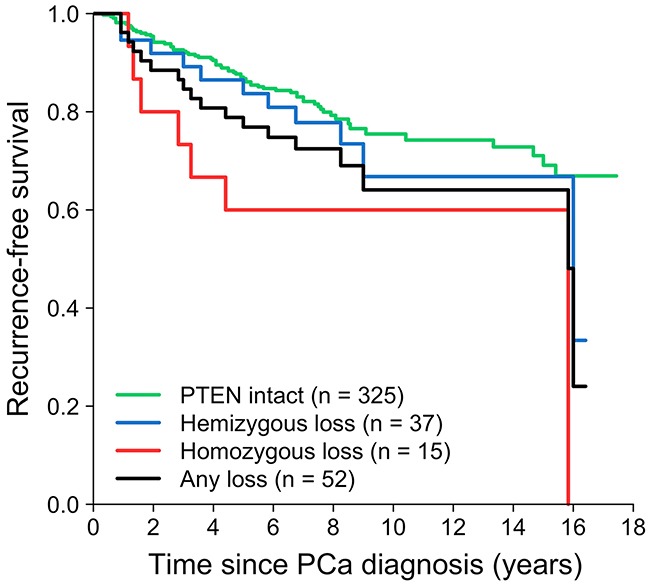
Loss of *PTEN* in relation to recurrence-free survival in the radical prostatectomy cohort

The association between *PTEN* loss and recurrence-free survival was investigated (Figure 1, Table 2). In total, 87 patients developed PCa recurrence during a mean follow-up of 8 years (Table 1). Compared to patients with *PTEN* intact tumors, those with homozygous *PTEN* deleted tumors had an increased risk of recurrence (HR = 2.84, 95% CI: 1.30, 6.19). Hemizygous loss was not significantly associated with recurrence. For any versus no *PTEN* loss, the HR was 1.74 (95% CI: 1.03, 2.93). The median time to recurrence in men with intact *PTEN*, hemizygous *PTEN* loss, and homozygous *PTEN* loss was 7.3, 7.8, and 5.7 years (P = 0.5), respectively.

**Table 2:** Age-adjusted hazard ratios and 95% confidence intervals for the association of *PTEN* loss with prostate cancer recurrence and by selected disease features

Patients	No. patients	No. events	*PTEN* status						
			Intact (ref.)	Hemizygous loss		Homozygous loss		Any loss	
			HR	HR	(95% CI)	HR	(95% CI)	HR	(95% CI)
All	377	87	1.00	1.39	(0.73, 2.64)	**2.84**	**(1.30, 6.19)**	**1.74**	**(1.03, 2.93)**
Local pathological stage	270	41	1.00	1.96	(0.86, 4.50)	**4.30**	**(1.31, 14.18)**	**2.35**	**(1.15, 4.83)**
Regional pathological stage	107	46	1.00	0.87	(0.31, 2.45)	1.23	(0.43, 3.49)	1.02	(0.47, 2.21)
Lower Gleason score (≤7)	318	58	1.00	1.23	(0.53, 2.89)	2.17	(0.78, 6.03)	1.49	(0.75, 2.96)
Higher Gleason score (8–10)	59	29	1.00	0.80	(0.26, 2.46)	**5.95**	**(1.58, 22.50)**	1.28	(0.51, 3.21)
*TMPRSS2:ERG* fusion-negative	161	37	1.00	2.26	(0.78, 6.54)	–	–	2.05	(0.70, 6.00)
*TMPRSS2:ERG* fusion-positive	198	45	1.00	1.20	(0.53, 2.72)	**3.26**	**(1.43, 7.45)**	1.75	(0.93, 3.29)

Subgroup analyses revealed significant associations between homozygous *PTEN* loss and PCa recurrence for patients with local pathological stage (HR = 4.30), higher Gleason score (8–10) tumors (HR = 5.95), and *TMPRSS2:ERG* fusion-positive tumors (HR = 3.26; Table 2). The association was not studied in the subgroup of patients with *TMPRSS2:ERG* fusion-negative tumors because only one patient in this subgroup had homozygous *PTEN* loss.

An ROC analysis revealed that a clinical model based on Gleason score and pathological stage had an AUC for PCa recurrence of 0.72. Loss of *PTEN* only had an AUC for recurrence of 0.69. After additionally including *PTEN* loss in the multivariable model with Gleason score and stage, the AUC improved slightly (0.73). Larger AUC improvements were observed in patients with local pathological stage (+3%), and in patients with higher Gleason scores of 8–10 (+4%).

### *PTEN* loss and tumor DNA methylation levels

**Figure 2:**
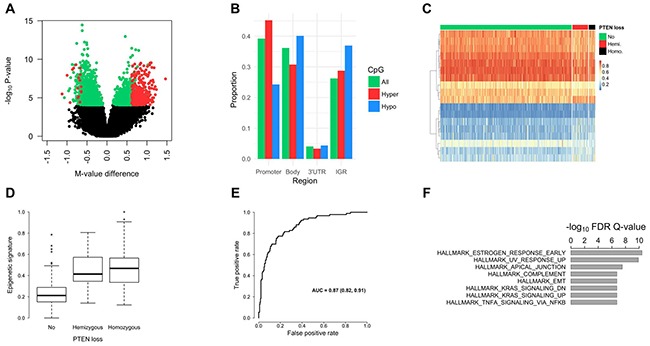
Prostate tumor DNA methylation profiles by *PTEN* status **(A)** Volcano plot for the differential methylation analysis of any *PTEN* loss versus intact *PTEN*. Each point in the figure represents a CpG site. Differentially methylated CpGs are shown in *green* or *red* (FDR Q-value < 0.01; n = 4,208). The CpGs shown in *red* have a mean methylation difference (*PTEN* loss vs. intact *PTEN*) of more than 10% (n = 485). CpGs with a higher mean methylation level in *PTEN* deleted tumors (i.e., hypermethylated) have a positive methylation M-value (logit transformation of β-value) difference, and hypomethylated CpGs have a negative M-value difference. **(B)** Proportion of significantly hypermethylated *(red bars)* and hypomethylated CpGs *(blue bars)* by genomic region. As a comparison, the proportion of all measured CpGs (n = 480K) by genomic region is shown *(green bars)*. **(C)** Heat map (supervised) of the 18 CpG sites selected using Elastic Net in our cohort. This panel of 18 CpGs optimally distinguished *PTEN* deleted from *PTEN* intact tumors. The rows of the heatmap are the CpG sites and the columns are the tumor samples, which were grouped based on *PTEN* status. Methylation β-values (figure legend; *range 0−1)* were used and the highest methylation levels are shown in *red*. The number of patients with intact *PTEN*, hemizygous *PTEN* loss, and homozygous *PTEN* loss was 388, 46, and 19, respectively. The rows were clustered based on Euclidean distance. **(D)** Epigenetic signature of *PTEN* loss in TCGA. The 18 differentially methylated CpGs, identified in our cohort, were combined into a single epigenetic signature, which was then tested in the TCGA dataset. As expected, tumors with *PTEN* loss had significantly higher levels of the signature compared to *PTEN* intact tumors. **(E)** ROC curve for classifying any *PTEN* loss versus intact *PTEN* using the methylation signature in TCGA. Values for the AUC and associated 95% confidence interval are shown in the figure. **(F)** Top-ranked GSEA hallmark gene sets, which showed enrichment for the genes with differentially methylated CpGs (1,908 genes).

Tumor epigenome-wide methylation differences between *PTEN* deleted (any loss) and *PTEN* intact tumors were studied. In total, 4,208 differentially methylated CpGs were identified (False Discovery Rate [FDR] Q-value < 0.01), of which 1,924 (46%) were hypermethylated in *PTEN* deleted tumors (Figure 2A). Of the 4,208 differentially methylated CpGs, 485 had a mean methylation difference of more than 10%. Genome-wide methylation levels in homozygous versus hemizygous *PTEN* deleted tumors were also compared. This analysis, however, revealed no significant differentially methylated CpGs between the two subsets (FDR Q-value = 1). Therefore, further methylation analyses involved contrasting tumors with any *PTEN* loss versus those with intact *PTEN*. Figure 2B shows the proportion of differentially methylated CpGs by genomic region, which showed that hypermethylated CpGs were more commonly found in gene promoter regions; and hypomethylated CpGs were more commonly found in gene body and intergenic regions.

The Elastic Net method was used to identify a panel of CpGs that, in combination, distinguished *PTEN* deleted from *PTEN* intact tumors in our cohort. In total, eighteen CpGs were identified (Table 3; Figure 2C). These CpGs were in 13 genes: *ATP11A, BAT4, CALD1, CSNK2B, GDNF, GNB1, JAK1, JAM3, RHOBTB1, RNF144A, SEZ6, VAPA, and YPEL3*; several of which have previously been implicated in cancer development and progression. The 18 CpGs were combined into a signature, as described in the methods. This signature was tested in the PCa TCGA dataset, which showed that both hemi- and homozygous deleted tumors have significantly higher levels of the signature compared to *PTEN* intact tumors (Figure 2D; P < 0.001). An ROC analysis showed an AUC of 0.87 for any *PTEN* loss versus intact *PTEN* (Figure 2E). The classification performance was similar in patient subsets based on tumor *TMPRSS2:ERG* fusion status.

**Table 3:** Eighteen top-ranked CpG sites for classifying prostate tumors with any *PTEN* loss versus intact *PTEN*

CpG ID	Chr.	Gene name	Genetic location	Epigenetic location	Mean β *PTEN* intact	Mean β *PTEN* deleted	Mean β difference	Elastic Net coefficient
cg05877648	6			Island	0.09	0.12	0.03	2.30
cg12150066	1	*GNB1*	TSS1500	S_Shore	0.09	0.14	0.05	1.16
cg17422460	6	*BAT4;CSNK2B*	Body;TSS1500	N_Shore	0.23	0.32	0.08	0.90
cg04121624	10	*RHOBTB1*	Body;TSS200	N_Shore	0.27	0.36	0.08	0.32
cg12444684	1	*JAK1*	5’UTR	N_Shore	0.17	0.27	0.10	0.13
cg27106909	16	*YPEL3*	1stExon;5’UTR	N_Shore	0.18	0.27	0.09	0.06
cg16166160	6			Island	0.18	0.26	0.08	0.05
cg03640071	11	*JAM3*	3’UTR		0.69	0.76	0.07	0.01
cg12930882	5	*GDNF*	Body		0.69	0.62	0.08	-0.10
cg20554353	7			S_Shore	0.79	0.72	0.07	-0.14
cg02072532	14				0.87	0.79	0.07	-0.31
cg13657981	7	*CALD1*	Body		0.79	0.73	0.06	-0.31
cg16937410	3				0.60	0.51	0.09	-0.73
cg20670923	18	*VAPA*	Body	S_Shore	0.41	0.34	0.07	-0.85
cg10162251	2	*RNF144A*	5’UTR		0.89	0.84	0.04	-0.90
cg04838191	2				0.87	0.81	0.06	-0.90
cg20708856	13	*ATP11A*	Body	S_Shore	0.84	0.77	0.07	-0.92
cg24742298	17	*SEZ6*	Body	N_Shelf	0.80	0.72	0.08	-1.47

### Pathway analysis

The 4,208 differentially methylated CpGs in *PTEN* deleted versus *PTEN* intact tumors were in 1,908 genes. Gene Set Enrichment Analysis (GSEA) showed that this gene list was enriched for genes in different pathways related to signaling, DNA repair, immune functions, and developmental processes (Figure 2F). Previously, Vivanco and colleagues identified gene expression differences in *PTEN* wild-type versus *PTEN* knockdown cell lines [28]. Epidermoid carcinoma, non-small-cell lung carcinoma, and mammary adenocarcinoma cells were used to generate the *PTEN* knockdown cell lines by performing retroviral transduction with a small hairpin RNA targeting *PTEN*. Comparing our findings to the sets of differentially expressed genes after *PTEN* knockdown *in vitro* (Molecular Signatures Database gene sets: PTEN_DN.V1_DN, PTEN_DN.V1_DN) showed significant gene set enrichment (GSEA, FDR Q-value < 0.0001). As such, these findings provide further evidence that the differentially methylated genes found in our study are at least partially regulated by *PTEN*. One of the upregulated genes after *PTEN* knockdown *in vitro* was *JAM3*, which was also identified in our methylation signature of *PTEN* loss. Further, Ouyang *et al*. studied gene expression differences in prostate tissue from *PTEN* mutant mice (Nkx3.1; Pten) [29]. One of the 20 upregulated genes in that study (gene set: OUYANG_PROSTATE_CANCER_PROGRESSION_UP) was *JAK1*, which is also one of the 18 genes included in the methylation signature of *PTEN* loss.

## DISCUSSION

This prospective study showed a positive association between homozygous *PTEN* loss and PCa recurrence after radical prostatectomy for clinically localized PCa. To gain further insights into the mechanisms that contribute to tumor progression in PCa patients with genomic deletion of *PTEN*, tumor DNA methylation profiles were investigated. The study revealed significantly different genome-wide methylation profiles in tumors classified by *PTEN* status, and identified a methylation signature that was uniquely associated with *PTEN* loss. The differentially methylated CpG sites were in biological pathways related to cell signaling (e.g., estrogen), DNA repair, and immune processes.

Several studies have shown that *PTEN* loss is associated with worse recurrence-free survival [5–16]. While some studies found that both homozygous and hemizygous loss increase the risk of adverse outcomes, two large, recent studies suggest a stronger association for homozygous loss. A study by Lotan *et al.* showed that patients with hemizygous and homozygous deleted tumors had a relative risk for recurrence of 1.24 (95% CI: 0.93, 1.65) and 1.66 (95% CI: 1.22, 2.24), respectively [12]. A study by Ahearn *et al.* evaluated the association of *PTEN* loss with PCa mortality and found that homozygous (HR = 1.9), but not hemizygous *PTEN* loss was significantly associated with a worse prognosis [5]. This suggests that tumors with a higher mass of *PTEN*-null cells have a higher propensity for metastatic spread [12].

The study by Lotan *et al.* also compared *PTEN* status to standard clinical-pathological parameters for predicting PCa recurrence (e.g., Gleason score, tumor stage) [12]. This showed that adding data on *PTEN* loss to the standard clinical model only modestly improved the AUC for recurrence (0.72 vs. 0.74). A similar result was seen in our study (0.72 vs. 0.73). However, predictors that result in small shifts in the AUC may be clinically useful and can improve clinical decision making for individual patients. Further, molecular tumor markers such as *PTEN* status might be more important and result in larger AUC improvements when detected in biopsy specimens from patients for whom data on pathological stage are unavailable.

Experimental studies have shown that tumor somatic *PTEN* loss in combination with the *TMPRSS2:ERG* gene fusion may result in accelerated tumor progression [18, 19]. The gene fusion exists in about half of all localized tumors in Caucasian men and is therefore the most common somatic alteration in PCa [6]; but presence of the fusion alone is not associated with adverse patient outcomes [30]. Several epidemiological and clinical investigations have studied the association between *PTEN* loss and adverse PCa outcomes in subgroups stratified by *TMPRSS2:ERG* fusion status, and the results are mixed. While some studies, including our study, found a stronger association of *PTEN* loss with recurrence among patients with *TMPRSS2:ERG* fusion-positive tumors [12, 15, 16], other studies reported a stronger association with prognosis in the *TMPRSS2:ERG* fusion-negative subgroup [5, 8, 10]. Importantly, one of the largest studies on *PTEN* loss and PCa recurrence to date by Lotan and coworkers [12], showed that *PTEN* loss was more strongly associated with recurrence-free survival among patients that harbored the gene fusion; but the authors also noted that there was no statistically significant interaction between *PTEN* loss and *TMPRSS2:ERG* fusion status. Thus, further research on this topic in larger datasets is needed.

Tumor DNA methylation profiling in our study revealed that tumors with hemi- and homozygous *PTEN* loss harbor significant genome-wide methylation alterations compared to *PTEN* intact tumors. These differentially methylated CpGs were enriched in genes involved in different biological processes such as signaling, DNA repair, immune functions, and developmental processes. The study also showed that many of the significant CpGs were in genes known to be differentially expressed after *PTEN* knockdown with RNAi, suggesting that these genes might be epigenetically regulated in PCa. One of the most significantly enriched pathways was related to estrogen signaling. Interestingly, previous research found that somatic *PTEN* mutations occur more frequently in tumors with estrogen receptor overexpression [31], and that estrogen receptor β (ERβ) is targeted for repression in PCa caused by *PTEN* deletion [32]. Our study also showed that DNA methylation profiles were similar in homozygous versus hemizygous deleted tumors. Thus, although some epidemiological, studies including our study, showed that patients with homozygous loss have a worse prognosis than patients with hemizygous loss, these prognostic differences appear to be unrelated to any substantial methylomic changes.

Using feature selection, we identified an 18-CpG methylation signature that classified tumors with *PTEN* loss. Importantly, this molecular classifier was validated using TCGA data where it accurately distinguished *PTEN* deleted from *PTEN* intact tumors. As the methylation signature is a genomic correlate of *PTEN* loss, the CpGs/genes included in the signature may provide mechanistic insights into the pathways altered in *PTEN* deleted tumors that contribute to PCa progression. The 13 genes (18 CpGs) in the *PTEN* signature have roles in various pathways, including cell signaling; and some of the genes have known roles in cancer development (e.g., *JAK1*, *GDNF*). Several of the genes have also been implicated in PCa or *PTEN* biology. For example, *VAPA* is an endogenous RNA that regulates *PTEN* levels in a microRNA-dependent manner [33].

Other noteworthy genes with CpGs in the epigenetic signature include *ATP11A*, *JAM3*, and *GDNF*. A previous study from our group identified a CpG biomarker in *ATP11A* for predicting metastatic-lethal PCa [26]. The gene encodes a membrane ATPase. *JAM3* was also included in the tumor mRNA expression signature of *PTEN* loss in breast cancer generated by Saal *et al.* [21], thereby providing further evidence of a link between this gene and *PTEN* activity. Aberrant methylation of *JAM3* has also been associated with cervical cancer [34]. Finally, *GDNF* has been shown to be elevated in PCa reactive tumor stroma and, as such, may contribute to tumor growth and invasion [35]. Therefore, for several of the genes that encompass CpGs in the signature there is plausible evidence for a role in prostate tumorigenesis.

Important strengths of the present study include the relatively large sample size and long-term follow-up for recurrence. Our methylation findings were confirmed using data from TCGA. A potential limitation of the study is that only a small subset of patients with PCa recurrence progressed to metastatic-lethal PCa so this critical endpoint could not be analyzed separately.

In conclusion, *PTEN* loss in PCa was associated with significantly altered epigenome-wide tumor methylation profiles. As PCa with *PTEN* loss has a more aggressive phenotype with shorter relapse-free survival, our findings suggest that aberrant DNA methylation may mediate tumor progression when *PTEN* is deleted.

## MATERIALS AND METHODS

### Study population

The cohort includes 566 PCa patients who underwent radical prostatectomy as primary therapy for clinically localized adenocarcinoma of the prostate. These patients were previously enrolled in population-based studies of PCa among residents of King County, WA (diagnosed in 1993–1996 or 2002–2005) [36, 37]. Clinical information and survival data were collected from the Seattle-Puget Sound Surveillance, Epidemiology, and End Results (SEER) Program cancer registry. Prostate cancer recurrence status was determined from two detailed follow-up surveys that were completed by patients in 2004–2005 and in 2010–2011, with review of medical records or physician follow-up as needed. A patient was considered to have disease recurrence based on: 1) a post-surgery PSA value of 0.2 ng/mL or greater; 2) metastatic progression on a bone scan, MRI, CT or biopsy; or 3) PCa-specific death. The mean follow-up time for biochemical recurrence was 8 years. The Fred Hutchinson Cancer Research Center Institutional Review Board approved the study and all participants signed informed consent statements.

### Fluorescence in situ hybridization (FISH)

Loss of *PTEN* was assessed using a FISH assay as described previously [38]. Hemizygous *PTEN* loss was defined as a ratio of the total number of *PTEN* signals divided by the total number of signals from the chromosome 10 centromere (CEP10) ≤ 0.75. Homozygous *PTEN* loss was defined as *PTEN*/CEP10 ≤ 0.2. In total, 71 patients had missing data on *PTEN* status. An additional 24 patients had *PTEN* gain; and these patients were not considered in the present analyses. FISH was also used to determine *TMPRSS2:ERG* gene fusion status, as described previously [39].

### DNA isolation, methylation profiling, and data preprocessing

Formalin-fixed paraffin-embedded prostate tumor tissue blocks were obtained from radical prostatectomy specimens and used to make hematoxylin and eosin stained slides, which were reviewed by a PCa pathologist to confirm the presence and location of prostate adenocarcinoma. For each patient two 1-mm tumor tissue cores from the dominant lesion enriched with ≥ 75% tumor cells were taken for DNA purification. The RecoverAll Total Nucleic Acid Isolation Kit (Ambion/Applied Biosciences, Austin, TX) was used to extract DNA, which was then shipped to Illumina (Illumina, Inc., San Diego, CA) for methylation profiling.

Tumor DNA was bisulfite converted. The Infinium HumanMethylation450 BeadChip array (Illumina) was used for methylation profiling. Methylation data were normalized using subset-quantile within array normalization (*minfi* in Bioconductor) [40], and batch effects were removed using ComBat [41]. Methylation β-values were calculated, which represent the percentage of DNA methylation at a CpG site. Methylation M-values were also calculated, which are a logit transformation of the β-values [42].

Genome annotation was based on the Illumina protocol. A gene promoter region was defined as: TSS1500, TSS200, 5’UTR, and 1stExon. Across the 96-well plates, we incorporated blind duplicate (n = 16) and replicate (n = 2) samples. A sample was excluded if less than 95% of the CpG sites for that sample on the array were detected with a detection P-value (probability of a CpG being detected above the background level defined by negative control probes) < 0.05. Further, CpG sites with a detection P-value of > 0.01 were excluded. Correlation coefficients for duplicate samples were 0.96–0.99. The correlation coefficient for the replicate samples was 0.99. After data preprocessing, there were 523 patients in the radical prostatectomy cohort with DNA methylation data.

### The cancer genome atlas (TCGA)

The TCGA PCa dataset included 333 patients, with oversampling of men with higher Gleason score tumors [43]. Allelic copy number derived from ABSOLUTE was used [44], along with relative copy number to determine hemizygous and homozygous *PTEN* deletions, as described previously [43]. In total, there were 50 homozygous *PTEN* deleted tumors, 43 hemizygous *PTEN* deleted tumors, and 240 *PTEN* intact tumors. Level 1 Infinium HumanMethylation450 data from TCGA (https://gdc.cancer.gov) were preprocessed as described above.

### Statistical data analysis

Cox proportional hazards regression and Kaplan-Meier analyses (*survival* in R) were used to evaluate the association of *PTEN* loss with PCa recurrence. In addition to the overall association, analyses were stratified by pathological stage, Gleason score, and *TMPRSS2:ERG* fusion status. Hazard ratios (HRs) and 95% confidence intervals (CIs) were computed. A ROC (Receiver Operating Characteristic) analysis was performed (*pROC* in R) to compare the prognostic classification performance (recurrence vs. no recurrence) of *PTEN* loss versus Gleason score (6, 7[3+4], 7[4+3], or 8–10) and pathological stage (local: pT2, N0/NX, M0; regional: pT3–T4 and/or N1, M0).

Epigenome-wide tumor methylation data (478,998 CpG sites) were analyzed to find differential methylation profiles between *PTEN* deleted (any *PTEN* loss) and *PTEN* intact tumors, and between hemi- and homozygous deleted tumors. Differentially methylated CpGs were identified using linear models (*limma* in Bioconductor).

Elastic Net regularization (*glmnet* in R; [45]) was used to identify a reduced panel of CpGs that, in combination, distinguished prostate tumors based on *PTEN* status. All measured CpG sites were used as input for the limma and glmnet analyses except CpGs in 10q22.1–10q25.1 (n = 7,604), which were excluded because this genomic region is deleted in tumors with *PTEN* loss [46]. Five-fold cross-validation and the AUC (Area Under the Curve) criterion were used to determine the optimal tuning parameter for classification. After variable selection using Elastic Net, the selected CpGs were combined into an epigenetic signature as follows: *signature_i_* = Σ_g=1_^n^ β_g_ × X_gi_, where g is the CpG site; n is the number of CpGs; β_g_ is the Elastic Net coefficient for CpG g; and X_gi_ is the methylation value for CpG g in the tumor of patient i.

A heatmap of the data was generated using *pheatmap* in R. Gene Set Enrichment Analysis (GSEA) was done using the Molecular Signatures Database (http://software.broadinstitute.org/gsea) by comparing our findings to the hallmark [47], and oncogenic signatures (C6) gene sets. All other analyses were done using R.
